# Phototheranostics of Splenic Myeloid-Derived Suppressor Cells and Its Impact on Spleen Metabolism in Tumor-Bearing Mice

**DOI:** 10.3390/cancers14153578

**Published:** 2022-07-22

**Authors:** James D. Barnett, Jiefu Jin, Marie-France Penet, Hisataka Kobayashi, Zaver M. Bhujwalla

**Affiliations:** 1 Division of Cancer Imaging Research, The Russell H. Morgan Department of Radiology and Radiological Science, The Johns Hopkins University School of Medicine, Baltimore, MD 21205, USA; jbarne55@jhmi.edu (J.D.B.); jjin@jhmi.edu (J.J.); mpenet2@jh.edu (M.-F.P.); 2 Sidney Kimmel Comprehensive Cancer Center, The Johns Hopkins University School of Medicine, Baltimore, MD 21205, USA; 3 Molecular Imaging Branch, Center for Cancer Research, National Cancer Institute, US National Institutes of Health, Bethesda, MD 20814, USA; kobayash@mail.nih.gov; 4 Department of Radiation Oncology and Molecular Radiation Sciences, The Johns Hopkins University School of Medicine, Baltimore, MD 21205, USA

**Keywords:** photoimmunotherapy, MDSCs, cancer, metabolism, spleen

## Abstract

**Simple Summary:**

Myeloid-derived suppressor cells (MDSCs) create an immune suppressive tumor microenvironment making them an important target in cancer. Here we demonstrated, in proof-of-principle studies, the ability of near-infrared photoimmunotherapy (NIR-PIT) to visualize and selectively eliminate MDSCs that are significantly expanded in the spleen of tumor-bearing mice using an anti-Gr1 antibody conjugated to the NIR dye IR700. Characterization of spleen extracts, using ^1^H magnetic resonance spectroscopy (MRS), identified metabolic changes indicative of MDSC elimination.

**Abstract:**

(1) Background: MDSCs play an active role in the immune surveillance escape of cancer cells. Because MDSCs in mice are CD11b^+^Gr1^+^, near-infrared photoimmunotherapy (NIR-PIT) using the NIR dye IR700 conjugated to an MDSC-binding antibody provides an opportunity for targeted elimination of MDSCs. (2) Methods: The efficacy of Gr1-IR700-mediated NIR-PIT was evaluated in vitro using magnetically separated CD11b^+^Gr1^+^ MDSCs from spleens of 4T1-luc tumor-bearing (TB) mice. For in vivo evaluation, spleens of Gr1-IR700-injected 4T1-luc TB mice were irradiated with NIR light, and splenocyte viability was determined using CCK-8 assays. Metabolic profiling of NIR-PIT-irradiated spleens was performed using ^1^H MRS. (3) Results: Flow cytometric analysis confirmed a ten-fold increase in splenic MDSCs in 4T1-luc TB mice. Gr1-IR700-mediated NIR-PIT eliminated tumor-induced splenic MDSCs in culture. Ex vivo fluorescence imaging revealed an 8- and 9-fold increase in mean fluorescence intensity (MFI) in the spleen and lungs of Gr1-IR700-injected compared to IgG-IR700-injected TB mice. Splenocytes from Gr1-IR700-injected TB mice exposed in vivo to NIR-PIT demonstrated significantly lower viability compared to no light exposure or untreated control groups. Significant metabolic changes were observed in spleens following NIR-PIT. (4) Conclusions: Our data confirm the ability of NIR-PIT to eliminate splenic MDSCs, identifying its potential to eliminate MDSCs in tumors to reduce immune suppression. The metabolic changes observed may identify potential biomarkers of splenic MDSC depletion as well as potential metabolic targets of MDSCs.

## 1. Introduction

Immature myeloid cells (iMCs) in healthy organisms differentiate into mature granulocytes, macrophages and dendritic cells as crucial components of the innate immune response to infection. Contrastingly, under pathological stress such as chronic inflammation and cancer, iMCs are activated during dysregulated myelopoiesis in the bone marrow and in extramedullary sites such as the spleen, giving rise to tumor-promoting myeloid-derived suppressor cells (MDSCs) [1]. MDSCs are major contributors to tumor progression [2] and metastasis [3,4,5]. MDSCs inhibit immune surveillance and compromise host-immune recognition in the tumor microenvironment (TME) [6]. They are highly potent immunosuppressors that both directly and indirectly suppress cytotoxic T cell function through various mechanisms ranging from T cell receptor (TCR) nitration [7] to arginine degradation [8,9]. MDSC numbers directly correlate with cancer disease progression and outcomes in both mice and humans [10].

In mice, MDSCs are broadly characterized as CD11b^+^Gr1^+^ with two major cell subsets, granulocytic MDSCs (G-MDSCs, CD11b^+^Ly6C^low^Ly6G^+^) and monocytic MDSCs (M-MDSCs, CD11b^+^Ly6C^high^Ly6G^−^) [11,12]. Because the anti-Gr1 antibody (RB6-8C5) can bind to Ly6G and Ly6C epitopes, it has been used in strategies to deplete MDSCs in mouse models [13,14,15]. MDSC depletion using anti-Gr1 was found to effectively eliminate MDSCs in the bone marrow, spleen, peripheral blood and primary tumors in some tumor models [15], although hepatic MDSCs persisted due to replenishment from the bone marrow [16]. Alternative strategies to deplete MDSCs are clearly needed. Other therapeutic strategies to target MDSCs in mouse models include chemotherapeutic depletion [17], modulation of MDSC differentiation [18] and chemotaxis inhibition to prevent MDSC migration and recruitment [19,20]. More recently, MDSC-specific “peptibodies” were developed to successfully deplete both subsets of MDSCs in the blood, spleen and tumor of tumor-challenged mice, inhibiting tumor growth with minimal adverse reactions during a multi-dose regimen; however, bone marrow CD11b^+^Gr1^+^ iMC were not affected, resulting in replenishment of systemic MDSCs at day 3 following single-dose treatment conditions [21]. Near-infrared photoimmunotherapy (NIR-PIT) using IR700-conjugated anti-Gr1 presents an opportunity to specifically and effectively eliminate MDSCs in the spleen, tumors or in circulation using a localized approach because only the cell membrane where the antibody conjugate binds is damaged with NIR light exposure. Mechanistic changes that lead to cell death following exposure of IR700 to NIR light have been well characterized [22,23].

### Purpose

To demonstrate, in proof-of-principle studies, that NIR-PIT with an antibody-photosensitizer conjugate (APC) Gr1-IR700 can be used to detect and eliminate Gr1^+^ MDSCs in culture and in the spleen in vivo, and to identify metabolic changes associated with splenic depletion of MDSCs.

## 2. Materials and Methods

### 2.1. Synthesis of IR700-Conjugated Anti-Gr1 and IgG Monoclonal Antibodies

Water-soluble phthalocyanine dye IRDye 700DX NHS ester (IR700) was purchased from LI-COR Biosciences (Lincoln, NE, USA). Anti-Gr1 monoclonal antibody (clone: RB6-8C5) and monoclonal rat IgG2b,κ antibody (clone: LTF-2) were obtained from BioXCell (Lebanon, NH, USA). Antibody-IR700 conjugate synthesis was carried out using 2 mg of antibody first dispersed in 2 mL of PBS. Next, 211 μg of IR700 (108 nmol, 1 mM in DMSO) was added. The mixture was vortexed vigorously and then kept at 4 °C overnight. Removal of unconjugated IR700 using ultra-centrifugal filter units (Millipore Amicon Ultra-0.5, 30 kDa cutoff) yielded Gr1- and IgG-IR700. The concentration of antibody and the dye/protein ratio were determined spectroscopically by using a DU730 UV/Vis Spectrophotometer (Beckman Coulter, Brea, CA, USA) to measure the absorbance of the conjugate at 280 nm and 689 nm. The extinction coefficients were 210,000 M^−1^cm^−1^ for both antibodies at 280 nm and 165,000 M^−1^cm^−1^ for IR700 at 689 nm. A correction factor of IR700 at 280 nm was set to be 0.095. The antibody–dye ratio was 1:3.4.

### 2.2. Animal Procedures

Murine 4T1-luc mammary carcinoma cells were purchased from SibTech Inc. (Brookfield, CT, USA) and cultured in Dulbecco’s Modified Eagle Medium (DMEM, Sigma, St. Louis, MO, USA) supplemented with 10% fetal bovine serum (FBS, Sigma Aldrich) at 37 °C under standard humidified incubation atmosphere containing 5% CO_2_. Animal studies were performed in accordance with guidelines established by the Johns Hopkins University Animal Care and Use Committee and conformed to the Guide for the Care and Use of Laboratory Animals published by the NIH.

Four- to six-week-old female BALB/c mice were purchased from Charles River Laboratories (Wilmington, MA, USA). Upon arrival and prior to 4T1-luc cell implantation, the mice were acclimatized and housed for at least 1 week in a light- and temperature-controlled vivarium with ad libitum food and water access.

### 2.3. Splenic MDSC Isolation

Spleens of tumor-bearing mice were dissected, gently pressed through a 70-micron cell strainer using a 5 mL syringe plunger and flushed with DMEM containing 2% FBS into a conical tube under sterile conditions. The single-cell splenocyte suspension was lysed of red blood cells using 1X RBC lysis buffer for 6 min on ice, and then the solution was neutralized using 1X PBS. MDSCs were isolated through magnetic-activated cell sorting (MACS) using the EasySep Mouse MDSC (CD11b^+^Gr1^+^) Isolation Kit (StemCell Technologies, 19867, Vancouver, BC, Canada) according to the manufacturer’s instructions.

### 2.4. Flow Cytometric Analysis

The following monoclonal antibodies were used: anti-Gr1-PE (phycoerythrin) (clone: RB6-8C5, BioLegend, San Diego, CA, USA) and anti-CD11b-FITC (clone: M1/70, BioLegend). Approximately 1 × 10^6^ cells were suspended in FACS buffer (PBS containing 1% bovine serum albumin and 2 mM EDTA). Cells were incubated with fluorophore-conjugated antibodies in the dark at 4 °C for 30 min, washed, and then stained with LIVE/DEAD™ Fixable Far Red dye (Invitrogen, Eugene, OR, USA) following the manufacturer’s protocol. Flow analyses were performed using a FACS Calibur system (Becton Dickinson Immunocytometry Systems, San Jose, CA, USA). IgG-PE and IgG-FITC (BioLegend) were used to distinguish specific stained population. FlowJo V10 software (FlowJo, LLC, Ashland, OR, USA) was used to quantify the percentages of positive events.

### 2.5. Biodistribution of Gr1-IR700

Female BALB/c mice were subcutaneously inoculated in the fourth right mammary fat pad with 1.5 × 10^6^ 4T1-luc breast cancer cells. Mice were randomly assigned into three cohorts consisting of tumor-bearing (TB) and non-tumor-bearing (NTB) mice: TB-Gr1-IR700 (*n* = 3), TB-IgG-IR700 (*n* = 4) and NTB-Gr1-IR700 (*n* = 4). TB mice were injected intraperitoneally (i.p.) with 100 µg of Gr1-IR700 or IgG-IR700 between 3 and 4 weeks post inoculation. As an additional control, age-matched NTB mice were injected with 100 µg of Gr1-IR700. Fluorescence imaging was performed on anesthetized mice under isoflurane (2% in oxygen) using the Pearl Impulse Small Animal Imaging System (LI-COR Biosciences). In vivo images were acquired pre-injection and 24 h after injection. Mice were then immediately euthanized to acquire ex vivo images of the small intestine, liver, lungs, stomach, blood, spleen, kidney, heart and tumor. Fluorescence intensities were analyzed using Pearl Impulse Software, v2.0 (LI-COR Biosciences).

### 2.6. In Vitro NIR Photoimmunotherapy and Phototoxicity Assay

CD11b^+^Gr1^+^ splenocytes were immunomagnetically isolated from TB mice as previously described. Cells were then suspended in DMEM with 10% FBS to a density of 1.5 × 10^5^ cells/100 µL, incubated with different concentrations of Gr1-IR700 or IgG-IR700 for 1 h, then washed and suspended in media. For all NIR-PIT experiments, 1.5 × 10^5^ cells were seeded in quadruplets into 96-well plates and exposed to varying NIR light doses to determine phototoxicity and reagent specificity. A power density of 24.2 mW/cm^2^, measured using an optical power meter (PM100, Thorlabs, Newton, NJ, USA), was used for all cell studies. NIR irradiation was achieved using a light emitting diode (Marubeni, Santa Clara, CA, USA) at a peak wavelength of 690 nm with NIR irradiation of 4, 8 and 16 J/cm^2^ with respective exposure times of 2.75, 5.5 and 11 min. The cells were incubated at 37 °C for 20 min after NIR irradiation. Next, 12.5 μL of CCK-8 reagent (Dojindo, Rockville, MD, USA) was added to each well, and the cells were further incubated at 37 °C for 100 min. For controls, cells were incubated with IgG-IR700 or PBS. Absorbance measurements of cell media were recorded at 450 nm. The phototoxicity data were presented as mean ± standard error of mean (SEM) from at least three independent experiments, with four technical replicates per experiment.

Concentration escalation experiments were conducted using 0, 1, 5 and 10 µg/mL Gr1-IR700 with NIR irradiation of 16 J/cm^2^. Exposure escalation experiments were performed using 10 µg/mL Gr1-IR700 with NIR irradiation of 4, 8 and 16 J/cm^2^. The targeting specificity of Gr1-IR700-mediated PIT was evaluated using 5 µg/mL Gr1-IR700, IgG-IR700 or PBS with NIR irradiation of 16 J/cm^2^. To determine whether Gr1-IR700-mediated phototoxicity could be inhibited by an anti-Gr1 antibody, cells were pre-treated with a fivefold unconjugated anti-Gr1 antibody for 20 min before Gr1-IR700 was added.

### 2.7. In Vivo NIR-PIT of Mouse Spleens

Female BALB/c mice were inoculated with 1.5 × 10^6^ 4T1-luc cells subcutaneously in the fourth right mammary fat pad. At 3–4 weeks post inoculation, mice were randomly assigned into four groups: NTB (*n* = 5), TB control (Ctrl, *n* = 5), Gr1-IR700 without PIT (Dark, *n* = 5) or Gr1-IR700 with PIT (PIT, *n* = 5). Mice in the Dark and PIT groups were shaved, injected intraperitoneally with 100 µg of Gr1-IR700 and monitored for 48 h. The 48 h time point was selected for in vivo PIT to allow clearance of circulating Gr1-IR700. Localization of Gr1-IR700 in the peritoneal cavity was confirmed through in vivo fluorescence imaging. At 48 h post i.p. injection, mice in the PIT group were anesthetized and the left abdominal region where the spleen is located was NIR-irradiated at 420 J/cm^2^. A power density of 175 mW/cm^2^ was used for in vivo PIT with an exposure time of 40 min. Aluminum foil was used to shield the rest of the body. Four hours after NIR-PIT, mice from all groups were sacrificed and spleens were either freeze clamped for proton magnetic resonance spectroscopy (^1^H MRS) analysis or disaggregated for cell viability studies.

### 2.8. Cell Viability of NIR-PIT-Treated Mouse Spleens

Following NIR-PIT, spleens were disaggregated, RBCs lysed, and splenocytes were suspended in CCK-8 solution with supplemented DMEM. Five hundred thousand cells were seeded into 96-well plates and incubated at 37 °C under standard tissue culture conditions for 100 min. Absorbance measurements were recorded at 450 nm using an Epoch microplate spectrophotometer (Biotek Instruments, Winooski, VT, USA).

### 2.9. Dual-Phase Extraction and ^1^H MRS Analysis of NIR-PIT-Treated Mouse Spleens

Following Gr1-targeted-NIR-PIT of the spleen, spleens from the NTB (*n* = 5), Ctrl (*n* = 5), Dark (*n* = 5) and PIT (*n* = 5) groups were cryopulverized in liquid nitrogen for dual-phase extraction. Pulverized spleens were gently vortexed in 3 mL of ice-cold methanol in a glass tube, and then 6 mL of ice-cold chloroform was added. The tissue suspensions were sonicated (50% duty, 1-s pulse cycle) on ice for 3 min, and then 2 mL of water was added, followed by sonication for 5 s. Samples were stored at 4 °C overnight to allow water and lipid phase separation. The biphasic extracts were centrifuged at 4500 rpm for 35 min at 4 °C. Aqueous phase extracts were isolated, lyophilized overnight and resuspended in 650 µL of deuterated water (D_2_O) containing sodium trimethylsilyl propionate (TSP) as an internal standard. ^1^H MRS was performed using a Bruker Avance III 750 MHz (17.6T) MR spectrometer for aqueous-phase metabolite identification. Spectral acquisition and data processing were performed using TOPSPIN 3.5 software. Selective variable-size binning and water resonance exclusion at 4.7 ppm were performed using AMIX 4.0 (Bruker Biospin, Billerica, MA, USA). Metabolites were identified by their chemical shifts, coupling constant and unique splitting patterns using chemical shift assignments from the Biological Magnetic Resonance Data Bank. Spectral intensity for each chemical shift was normalized to the TSP reference peak and spleen sample weight. A heat map of aqueous-phase metabolites was generated using MATLAB software (MATLAB R2020b) to illustrate metabolic changes.

### 2.10. Statistical Analysis

Data were presented as mean ± SEM from at least three individual samples or mice. Statistical analysis was performed using a two-sided Student’s *t*-test (GraphPad Prism, San Diego, CA, USA). Values of *p* ≤ 0.05 were considered statistically significant unless otherwise stated.

## 3. Results

### 3.1. Splenic MDSCs Significantly Increased in 4T1-Luc Tumor-Bearing Mice

**Figure 1:**
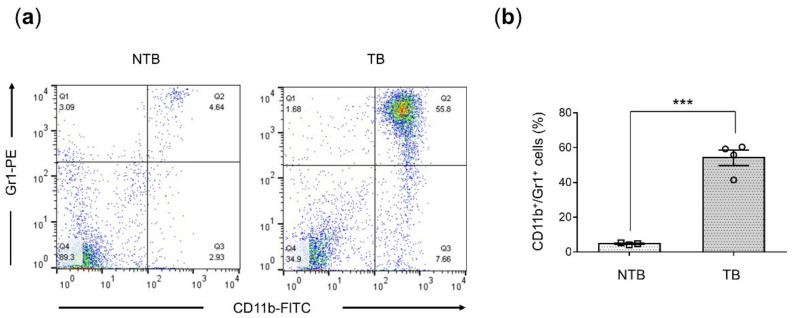
Accumulation of splenic MDSCs in tumor-bearing mice. (**a**) Representative flow cytometric analysis comparing CD11b and Gr1 expression in splenocytes from non-tumor-bearing (NTB) to 4T1-luc tumor-bearing (TB) four- to six-week-old female BALB/c mice at 3–4 weeks post-tumor implantation. (**b**) Percentage of CD11b^+^Gr1^+^ MDSCs in the spleen of NTB (*n* = 3) and TB (*n* = 4) mice. Values represent mean ± SEM. Two-tailed Student’s *t*-test. *** *p* ≤ 0.001.

The percentage of MDSCs in the spleen significantly increased (*p* = 0.0002) from 4.78 ± 0.3% in NTB mice (*n* = 3) to 54.3 ± 4.4% in their TB counterparts (*n* = 4) at 3–4 weeks post tumor implantation, as shown by representative flow cytometry data in Figure 1a and summarized in Figure 1b.

### 3.2. Gr1-IR700-Mediated PIT Eliminated Tumor-Induced MDSCs Isolated from the Spleen

**Figure 2:**
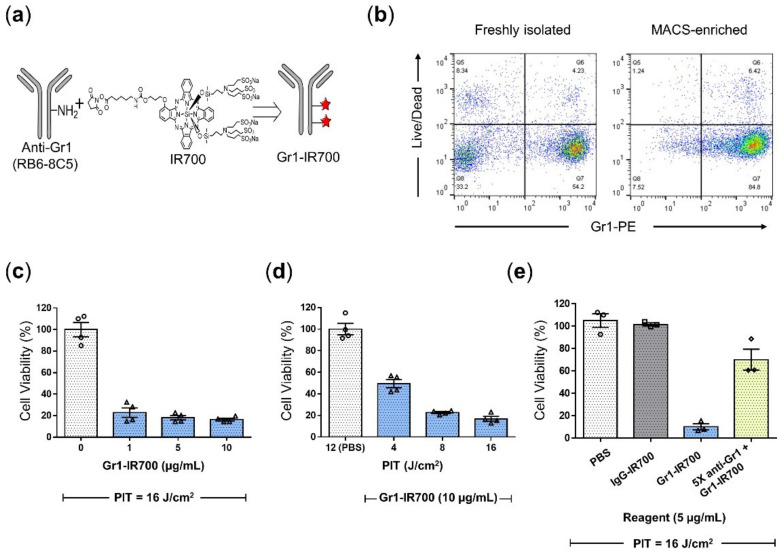
Gr1-IR700 eliminates tumor-induced splenic MDSCs in vitro. (**a**) Schematic of Gr1-IR700 synthesis. (**b**) Representative flow cytometric analysis of freshly isolated and MACS-enriched (84.5% ± 2.4%) Gr1^+^ splenocytes (*n* = 4). (**c**) Gr1-IR700-mediated PIT reduced cell viability at a fixed NIR light dose of 16 J/cm^2^ and increasing concentration of Gr1-IR700 (*n* = 4). (**d**) Gr1-IR700-mediated cell killing increased in a NIR-light dose-dependent manner (*n* = 4). (**e**) Selective Gr1-IR700-PIT-mediated cell killing compared to NIR-irradiated PBS and IgG-IR700 groups. Phototoxicity was partially inhibited after 5-fold excess pre-incubation using unconjugated anti-Gr1. Values represent mean ± SEM. Each of the figure panels represents at least 3 experimental replicates.

Synthesis of Gr1-IR700 was carried out through the attachment of NHS-activated IR700 to the free amine residues on the Gr1 antibody (clone: RB6-8C5), as shown in the schematic in Figure 2a. To evaluate the ability of NIR-PIT to eliminate CD11b^+^Gr1^+^ MDSCs, splenocytes from TB mice were immunomagnetically isolated. An MDSC purity of 84.5% was confirmed in MACS-enriched cells by flow cytometric analysis (Figure 2b) before NIR-PIT experiments were conducted. Concentration escalation experiments (Figure 2c) indicated a significant reduction in the viability of NIR-irradiated cells to 23%, 18% and 16% using 1, 5 and 10 µg/mL Gr1-IR700 compared to PBS. Photoirradiation-dose dependency was observed at a fixed concentration of 10 µg/mL Gr1-IR700 (Figure 2d). Only NIR-irradiated cells treated with Gr1-IR700 demonstrated reduced viability (Figure 2e) compared to PBS- and IgG-IR700-treated cells. A minor reduction in cell viability was observed in cells that were pre-incubated with a five-fold excess unconjugated anti-Gr1 antibody.

### 3.3. Gr1-IR700 Preferentially Accumulated in the Spleen and Lungs of Tumor-Bearing Mice

**Figure 3:**
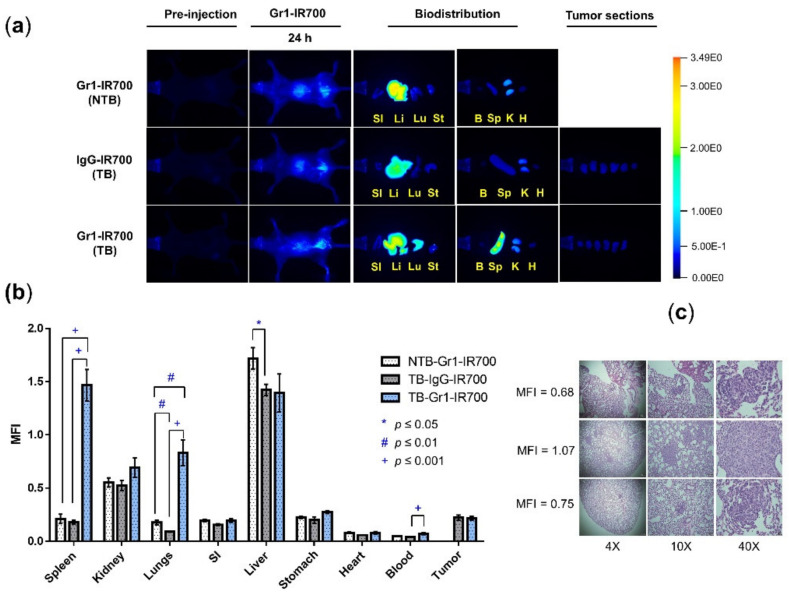
Gr1-IR700 preferentially accumulates in the spleens and lungs of TB mice. (**a**) Representative in vivo fluorescence images obtained before and after administration of Gr1-IR700 in NTB mouse (top), IgG-IR700 in TB mouse (middle), and Gr1-IR700 in TB mouse (bottom) and corresponding ex vivo fluorescence images of the small intestine (SI), liver (Li), lung (Lu), stomach (St), blood (B), spleen (Sp), kidney (K) and heart (H). Also included is ex vivo fluorescence from tumor slices at 1 mm thickness (right). (**b**) Biodistribution from Mean Fluorescence Intensity (MFI) of Gr1-IR700 and IgG-IR700 in Gr1-IR700-injected NTB mice (*n* = 4), IgG-IR700-injected TB mice (*n* = 4) and Gr1-IR700-injected TB mice (*n* = 3) at 24 h post-intraperitoneal injection. MFI values indicate significantly higher MFI in the spleen, lungs and blood of TB mice injected with Gr1-IR700. Two-tailed Student’s *t*-test. * *p* ≤ 0.05, ^#^ *p* ≤ 0.01, and ^+^ *p* ≤ 0.001. (**c**) Hematoxylin and eosin stained 5 µm thick lung sections from three Gr1-IR700-injected TB mice at 4X (left), 10X (middle) and 40X (right) magnification, with the corresponding MFI in the lung sections displayed adjacent to the 40X section. MFI was highest in the lungs with the highest number of nodules.

NIR images of mice were acquired after i.p. injection of Gr1-IR700 or IgG-IR700 in NTB or TB mice. Representative in vivo and ex vivo images are presented in Figure 3a. The retention of Gr1-IR700 is evident in the ex vivo images of the spleen and lungs of TB mice that was not observed in the TB-IgG-IR700 and the NTB-Gr1-IR700 mice. A significant increase in mean fluorescence intensity (MFI) was observed in the spleen, lungs and blood of TB-Gr1-IR700 mice compared to NTB-Gr1-IR700 and TB-IgG-IR700 mice, as shown in Figure 3b, identifying the recruitment and expansion of CD11b^+^Gr1^+^ MDSCs in the spleen and lungs of the TB group. Metastatic nodules were detected in the lungs of TB-Gr1-IR700 mice, as shown in Figure 3c. Lungs with the highest number of nodules also exhibited the highest MFI. The significant increase in MFI in the blood may reflect MDSCs in the circulation. We also observed non-specific accumulation of Gr1- as well as IgG-IR700 in the liver that may have been due to hepatic clearance or due to the presence of hepatic MDSCs in TB mice.

### 3.4. In Vivo Gr1-IR700-Mediated PIT of Tumor-Bearing Mouse Spleens Demonstrated Significant Reduction of Splenocyte Viability

**Figure 4:**
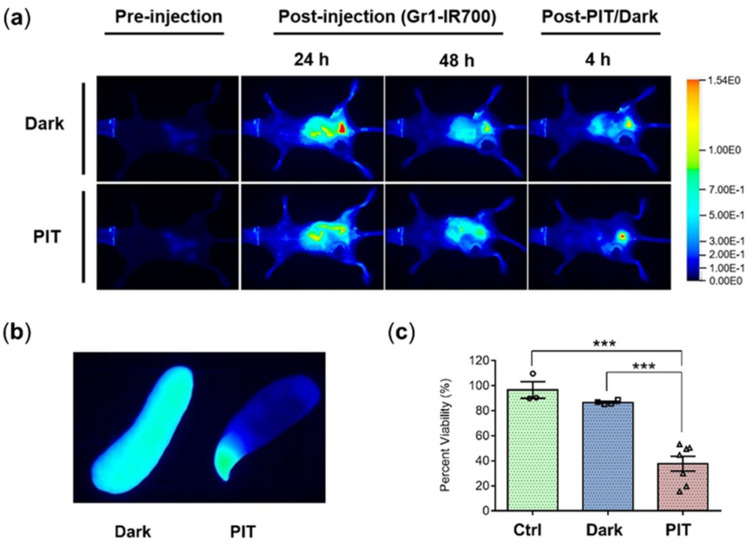
In vivo Gr1-IR700-mediated PIT of tumor-bearing mouse spleens. (**a**) Representative in vivo fluorescence images of mice pre-injection and at 24 h and 48 h post-IP injection of Gr1-IR700 without light (top) or exposed to NIR light (bottom). NIR-PIT was administered at an exposure dose of 420 J/cm^2^ with a red light-emitting diode (LED) at a peak wavelength of 690 nm. Representative images from mice that were further imaged at 4 h post-PIT are also shown. Dark and PIT correspondingly refer to non-irradiated TB mice and NIR-irradiated TB mice. Exposure to light resulted in a decrease in fluorescence due to the breakdown of IR700. (**b**) Representative ex vivo fluorescence image of excised mouse spleen from the Dark (left) and PIT group (right) confirmed the loss of IR700-fluorescence in the PIT-treated spleen due to NIR light exposure. (**c**) Cell viability of splenocytes measured using 5 × 10^5^ splenocytes from excised spleens of the uninjected TB Ctrl (*n* = 3), Dark (*n* = 4) and PIT (*n* = 7) groups incubated in CCK-8 solution for 90–100 min. Two-tailed Student’s *t*-test. *** *p* ≤ 0.001.

Following Gr1-IR700 injection, NIR imaging was performed to obtain in vivo images and ex vivo spleen images. Representative in vivo fluorescence images of mice pre-injection and at 24 h and 48 h post-IP injection of Gr1-IR700 without light (top) or exposed to NIR light (bottom) are presented in Figure 4a. Also shown in Figure 4a are representative images from mice imaged at 4 h post-PIT. Dark or PIT correspondingly refer to non-irradiated mice and NIR-irradiated mice. Exposure to light resulted in a decrease in fluorescence due to the breakdown of IR700. Representative ex vivo fluorescence images of excised mouse spleen from the Dark (left) and PIT group (right) confirmed the loss of IR700-fluorescence in the PIT-treated spleen due to NIR light exposure (Figure 4b). Although the liver was shielded with aluminum foil, NIR-PIT-induced damage to liver tissue may have occurred due to its close proximity to the spleen. A significant decrease in splenocyte viability was evident after NIR-PIT. The number of viable splenocytes was reduced from 90 ± 1.7% in the TB Dark group to 39.5% ± 6.3% in the TB PIT group compared to the untreated TB Ctrl group (Figure 4c).

### 3.5. In Vivo Gr1-IR700-Mediated PIT of Tumor-Bearing Mouse Spleens Resulted in Significant Metabolic Changes

**Figure 5:**
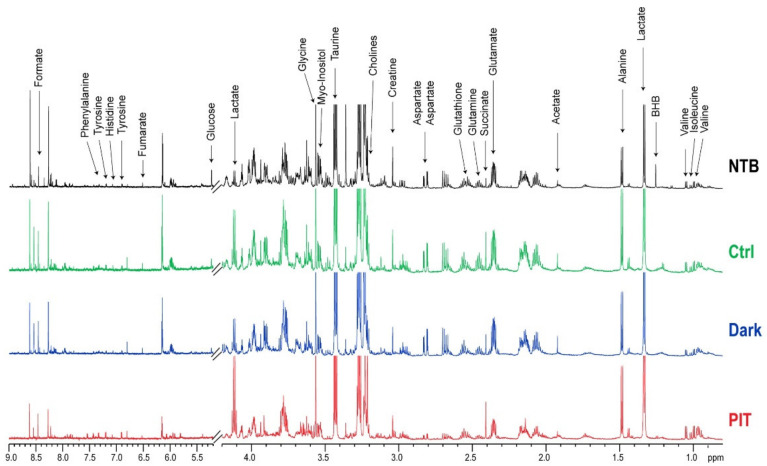
Representative ^1^H-MR spectra of aqueous spleen metabolites from NTB (black), tumor-bearing Ctrl (green), Gr1-IR700-injected Dark (blue) and Gr1-IR700-injected PIT (red) mice obtained 4 h post-PIT. BHB—beta-hydroxybutyrate; PC—phosphocholine; GPC—glycerophosphocholine.

**Figure 6:**
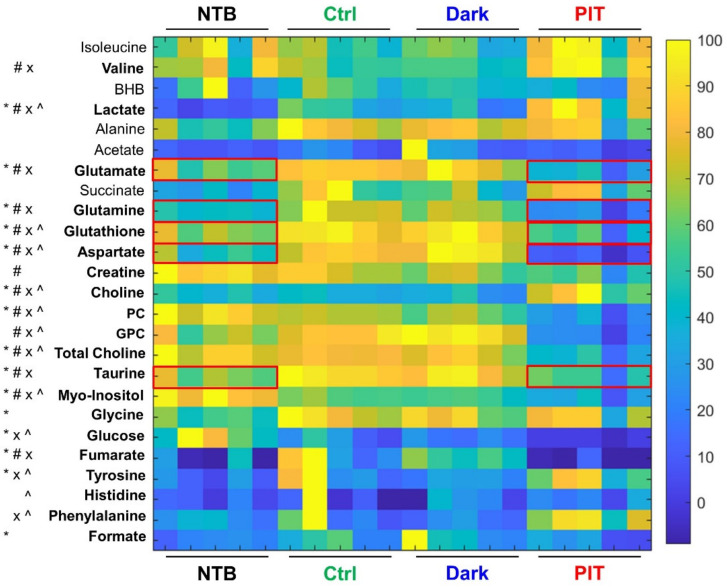
Heat map of aqueous spleen metabolites from NTB (*n* = 5), TB Ctrl (*n* = 5), Gr1-IR700-injected Dark (*n* = 5) and Gr1-IR700-injected PIT (*n* = 5) mice 4 h post-PIT. Bold font indicates significant percent changes with two-tailed Student’s *t*-test, *p* ≤ 0.05. * denotes NTB vs. Ctrl, + denotes Ctrl vs. Dark, # denotes Ctrl vs. PIT, x denotes Dark vs. PIT and ^ denotes NTB vs. PIT. BHB—beta-hydroxybutyrate; PC—phosphocholine; GPC—glycerophosphocholine. The red boxes denote metabolites in the spleen of TB mice that normalized to NTB levels following PIT.

Representative ^1^H-MR spectra from the spleens of NTB (black), TB Ctrl (green), TB Gr1-IR700-injected Dark (blue) and TB Gr1-IR700-injected PIT (red) mice obtained 4 h post-PIT presented in Figure 5, demonstrate the alterations in metabolic patterns in the spleens of TB Gr1-IR700-injected PIT mice. The results summarized in the heat map presented in Figure 6 demonstrate that metabolite levels of glutamate, glutamine, glutathione, aspartate, taurine and fumarate in the spleen of Gr1-IR700-injected PIT mice were normalized to levels comparable to NTB mice, whereas levels of these metabolites were comparable in spleens from the TB Ctrl group and the Gr1-IR700-injected Dark group. Gr1-IR700-PIT also resulted in a significant increase in lactate and choline and a significant decrease in phosphocholine (PC) and glycerophosphocholine (GPC). Metabolite concentrations presented in Table 1 summarize the metabolic changes that occurred in the TB Ctrl, TB Gr1-IR700-injected Dark and TB Gr1-IR700-injected PIT groups compared to the NTB group. There were no significant metabolic differences between the TB Ctrl and the TB Gr1-IR700-injected Dark groups.

## 4. Discussion

We found that the growth of 4T1-luc tumors significantly expanded the CD11b^+^Gr1^+^ MDSC population in the spleen from approximately 5% in NTB mice to approximately 54% in TB mice. A significant dose-dependent or light-exposure-dependent reduction of viability was observed in vitro following NIR-PIT of CD11b^+^Gr1^+^ MDSCs isolated from the spleen, confirming the ability of Gr1-IR700-PIT to eliminate MDSCs. In vivo biodistribution studies with NIR fluorescence imaging identified the highest Gr1-IR700 specific uptake in the spleen, followed by the lungs and peripheral blood. Metastatic nodules were detected in the lungs, with the highest Gr1 uptake observed in the lungs with the most metastatic nodules. Gr1-IR700 uptake was not significantly different from IgG-IR700 non-specific uptake in tumors. In vivo NIR-PIT of the spleens resulted in splenocyte viability dropping to 39.5% compared to the 90% cell viability in mice injected with Gr1-IR700 but not exposed to NIR light, and the 100% viability in TB untreated mice, consistent with the elimination of MDSCs in the spleen by NIR-PIT. Metabolic changes were identified between NTB and TB untreated and TB Gr1-IR700 groups as well as the TB Gr1-IR700-PIT group. Interestingly, several metabolites in the spleens of the NIR-PIT group normalized to metabolic levels observed in the NTB group.

Our observations that 4T1-luc tumor growth significantly expanded MDSCs are consistent with results from previous studies that have identified the recruitment and expansion of CD11b^+^Gr1^+^ MDSCs in the spleen and lungs during 4T1 tumor progression [24,25,26]. The significant retention of Gr1-IR700 in the spleens of TB mice, identified by significantly increased fluorescence intensity, was consistent with the expansion of MDSCs in the spleen with tumor growth. The Gr1-specific uptake in the lungs was also consistent with the role of MDSCs in tumor progression and metastasis [27]. MDSCs facilitate metastasis by orchestrating the ability of tumor cells to evade immune recognition. MDSCs, specifically G-MDSCs, have been identified as major contributors to the premetastatic niche formation in distal organs such as the lungs [27]. Although the role of MDSCs in circulating and disseminated tumor cell dynamics is not well understood, lungs with metastatic lesions of primary cancers are comprised of G-MDSCs with the potential to enhance metastatic growth [28]. The Gr1-specific uptake in peripheral blood may reflect the presence of circulating MDSCs in the TB mice. Elevated peripheral blood MDSCs are commonly observed in tumor-bearing mice [29] and cancer patients [30,31,32,33], with G-MDSC being the predominant immunosuppressive subset [34] and M-MDSCs proposed to be the more T-cell-suppressive subset [35]. In our studies, however, we did not observe a significant Gr1-specific uptake in the primary 4T1 tumor, which may reflect the far fewer MDSCs present in the primary tumor, unlike in the metastatic nodules. Gr1 expression levels, the number of Gr1-antibody binding cells and the amount of Gr1-antibody conjugate reaching the tumor are the three major determinants of fluorescence intensity. The lack of fluorescence signal in tumors is most likely due to a combination of all three factors. Previous studies found that at 3 weeks post-implantation of 4T1 cells, CD11b^+^Gr1^+^ cells comprised approximately 70% of the viable immune cells in the lungs, accompanied by a gradual increase in lung-infiltrating tumor cells [24].

Here, for the first time, we applied NIR-PIT using Gr1-IR700 to demonstrate the feasibility of eliminating MDSCs in culture and in the spleen in vivo. Although we did not detect increased Gr1 fluorescence in the tumors due to detection sensitivity limitations, our proof-of-principle studies demonstrate the potential of using such a strategy to eliminate MDSCs in primary and metastatic tumors to understand the role of MDSCs and their impact on the tumor microenvironment and metastasis in experimental mouse models. Exposure of circulating peripheral blood to a light source placed at a major vessel may be used to eliminate circulating MDSCs to reduce their ability to establish premetastatic niches.

Unlike murine MDSCs, subsets of human MDSCs have been less extensively studied. Selectively targeting human MDSCs remains challenging due to their heterogeneous nature and the lack of functional characterization. Human M-MDSCs are HLADR^−^CD11b^+^CD15^−^CD14^+^ and human G-MDSCs are HLADR^−^CD11b^+^CD15^+^CD14^−^CD66b^+^: myeloid differentiation markers CD14 and CD15 can distinguish the major human MDSC subsets [36]. The cell surface marker CD84 was identified through phenotyping as a robust marker of both human G-MDSCs and M-MDSCs that may provide a translational NIR-PIT approach [37]. In the clinical setting, NIR light can be administered externally to target MDSCs in superficial primary tumors or at a large superficial blood vessel, or endoscopically using fiber optic diffusers to eliminate MDSCs during the pre-metastasis window in patients with aggressive cancer subtypes. Cetuximab-IR700 (RM-1929), targeting epidermal growth factor receptor (EGFR), is now being evaluated for the treatment of inoperable head and neck squamous cell cancer (HNSCC) in a global phase III clinical trial [38]. The first APC for human use, a cetuximab-IR700 conjugate (AkaluxTM; Rakuten Medical Inc., San Diego, CA, USA), and a NIR laser system (BioBladeTM; Rakuten Medical Inc., San Diego, CA, USA) were approved for clinical use by the Pharmaceuticals and Medical Devices Agency in Japan in September 2020. NIR light has limited penetration. Newer methodologies that use X-ray excitation to generate NIR light may address this limitation in the future [39,40].

There is an increased interest in the role of metabolism in mediating the immune response to cancer. We found that spleen metabolism was significantly altered in TB mice compared to NTB. Since the presence of 4T1 tumors significantly expanded the MDSC population to more than 50%, some of these metabolic alterations may have been specific to the MDSC population. The metabolite levels of glutamate, glutamine, glutathione, aspartate, taurine and fumarate in the spleen of Gr1-IR700-injected PIT mice returned to levels comparable to NTB mice, suggesting that the increases of these metabolites observed in untreated TB mice and Gr1-IR700 injected mice not exposed to light were likely due to the MDSCs that subsequently were eliminated with NIR-PIT. The elevation of lactate in the spleens of PIT mice compared to all other groups may have been due to NIR-PIT-induced membrane lipid peroxidation, leading to reactive oxygen species (ROS) generation and necrotic cell death [41,42]. Elevated choline and reduced PC, GPC and total choline were indicative of disrupted choline metabolism and phospholipid membrane destruction by Gr1-IR700-PIT. We did not determine the effect of light alone on spleen metabolism that may have contributed to the effects observed here. PIT of TB mouse spleens resulted in a reduction of taurine comparable to NTB mice. Elevated taurine is associated with inflammation and increased oxidant concentrations [43,44]. Previous studies have reported enriched taurine and hypotaurine metabolism through proteomic analysis of splenic MDSCs isolated from 4T1-TB mice [45], further suggesting targeting of splenic MDSCs in our studies. Some of the metabolites that normalized following NIR-PIT are associated with T-cell dysfunction. An excess of extracellular glutamate in the TME was found to result in T-cell dysfunction [46], and taurine was reported to promote the production of FOXP3^+^ regulatory T cells that are immune suppressive [47]. Normalization of these metabolites may improve T-cell function and anti-tumor response following immune checkpoint therapies. The absence of any metabolic differences in the spleen between the TB mice and Gr1-IR700 injected mice confirmed that treatment with Gr1 alone did not alter spleen metabolism. Changes in the spleen metabolites following NIR-PIT may provide biomarkers to detect changes in expanded MDSC populations, as well as provide metabolic targets for MDSCs.

## 5. Conclusions

In summary, we have demonstrated the feasibility of eliminating MDSCs using NIR-PIT with Gr1-IR700. Future studies should evaluate the effects of Gr1-IR700 NIR-PIT on the metastatic ability of primary tumors. Translational efforts should investigate the feasibility of CD84-IR700 to eliminate human MDSCs with NIR-PIT. The metabolic changes observed here may provide biomarkers to detect changes in MDSC populations and identify metabolic targets for MDSCs.

## Figures and Tables

**Table 1:** ^1^H MRS analysis of spleen metabolite concentrations from non-tumor-bearing NTB (*n* = 5), TB Ctrl (*n* = 5), Gr1-IR700-injected Dark (*n* = 5) and Gr1-IR700-injected PIT (*n* = 5) mice 4 h post-PIT. Bold font indicates significant percent changes evaluated with two-tailed Student’s *t*-test comparing Ctrl vs. PIT, Dark vs. PIT, NTB vs. Ctrl and Ctrl vs. Dark. Values represent mean ± SEM, *n* = 5 per group. BHB—beta-hydroxybutyrate; PC—phosphocholine; GPC—glycerophosphocholine.

Metabolites	Chemical Shift (ppm)	Concentration (mM)				Percent Change (%), *p*-Value							
		NTB	Ctrl	Dark	PIT	Ctrl vs. PIT		Dark vs. PIT		NTB vs. PIT		Ctrl vs. Dark	
***Isoleucine***	1.02	0.101	0.084	0.079	0.116	38	0.078	47	0.052	15	0.459	−6	0.668
***Valine***	1.05	0.170	0.144	0.129	0.204	**41**	**0.018**	**57**	**0.003**	20	0.190	−10	0.275
***BHB***	1.20	0.127	0.151	0.136	0.125	−17	0.426	−8	0.708	−2	0.969	−10	0.399
***Lactate***	1.33	1.066	3.163	2.367	5.048	**60**	**0.024**	**113**	**0.004**	**374**	**1 × 10^−4^**	−25	0.160
***Alanine***	1.48	0.666	0.932	0.891	0.798	−14	0.312	−10	0.442	20	0.317	−4	0.524
***Acetate***	1.92	0.068	0.089	0.163	0.065	−27	0.214	−60	0.143	−5	0.771	82	0.264
***Glutamate***	2.35	3.247	4.391	4.264	1.962	**−55**	**3 × 10^−5^**	**−54**	**4 × 10^−4^**	**−40**	**0.008**	−3	0.652
***Succinate***	2.41	0.104	0.196	0.149	0.187	−5	0.794	26	0.233	**80**	**0.011**	−24	0.162
***Glutamine***	2.46	0.922	1.516	1.311	0.500	**−67**	**7 × 10^−5^**	**−62**	**5 × 10^−5^**	**−46**	**0.001**	−13	0.136
***Glutathione***	2.54	3.801	4.979	4.905	2.629	**−47**	**0.001**	**−46**	**0.002**	**−31**	**0.037**	−1	0.809
***Aspartate***	2.80	0.437	0.675	0.721	0.120	**−82**	**7 × 10^−8^**	**−83**	**1 × 10^−6^**	**−72**	**2 × 10^−4^**	7	0.347
***Creatine***	3.04	0.380	0.334	0.291	0.227	**−32**	**0.010**	−22	0.083	**−40**	**0.001**	−13	0.100
***Choline***	3.21	0.155	0.142	0.129	0.249	**75**	**0.004**	**93**	**0.004**	**61**	**0.010**	−9	0.433
***PC***	3.23	1.903	1.606	1.437	0.680	**−58**	**3 × 10^−5^**	**−53**	**5 × 10^−4^**	**−64**	**5 × 10^−5^**	−10	0.072
***GPC***	3.24	1.261	1.550	1.670	0.478	**−69**	**2 × 10^−6^**	**−71**	**3 × 10^−6^**	**−62**	**9 × 10^−5^**	8	0.203
***Total Choline***	3.23	3.345	3.215	3.088	1.585	**−51**	**8 × 10^−5^**	**−49**	**5 × 10^−4^**	**−53**	**3 × 10^−4^**	−4	0.446
***Taurine***	3.43	9.036	12.408	11.58	6.820	**−45**	**0.001**	**−41**	**0.004**	−25	0.087	−7	0.253
***Myo-Inositol***	3.55	2.777	1.964	1.770	1.131	**−42**	**0.002**	**−36**	**0.009**	**−59**	**6 × 10^−5^**	−10	0.082
***Glycine***	3.56	0.649	0.951	0.877	0.836	−12	0.371	−5	0.757	29	0.136	−8	0.464
***Glucose***	5.24	0.140	0.063	0.055	0.016	**−75**	**0.015**	**−71**	**9 × 10^−5^**	**−89**	**4 × 10^−4^**	−13	0.633
***Fumarate***	6.52	0.010	0.033	0.034	0.003	**−92**	**0.015**	**−92**	**1 × 10^−5^**	−75	0.281	3	0.930
***Tyrosine***	6.90	0.034	0.078	0.046	0.098	26	0.461	**110**	**5 × 10^−3^**	**187**	**0.002**	−40	0.214
***Histidine***	7.08	0.031	0.051	0.041	0.048	−6	0.930	16	0.703	55	0.128	−19	0.795
***Phenylalanine***	7.43	0.030	0.040	0.022	0.066	65	0.133	**196**	**5 × 10^−4^**	**119**	**0.002**	−44	0.242
***Formate***	8.46	0.066	0.101	0.122	0.066	−35	0.143	−46	0.137	0	0.987	21	0.570

## Data Availability

The data presented in this study are available on request from the corresponding author.
